# Radial artery as a second conduit gains momentum: The RAPCO trial

**DOI:** 10.21542/gcsp.2021.10

**Published:** 2021-06-30

**Authors:** Walid Simry, Ahmed Afifi

**Affiliations:** 1 Cardiac Surgery Department, Aswan Heart Centre, Aswan, Egypt

## Abstract

In coronary artery bypass grafting (CABG), the use of an internal mammary artery (IMA) to graft the left anterior descending coronary artery (LAD) improves survival and reduces the need for repeat revascularization. The other IMA, radial artery (RA), and saphenous vein (SV) have contested to complete the surgical revascularization. For that purpose, SV remains the most commonly used conduit despite current evidence in favor of arterial grafts. To determine which conduit is best for grafting the second most important coronary artery, Buxton and colleagues have recently published the long term results of their “Radial Artery Patency and Clinical Outcomes (RAPCO)” trial.

## Introduction

Coronary artery bypass grafting (CABG) surgery is the best treatment modality for ischemic heart disease (IHD) patients^1^. The use of an internal mammary artery (IMA) to graft the left anterior descending coronary artery (LAD) has long been proven to improve survival and reduce the need for repeat revascularization^2^. It is not similarly clear which is the best conduit to use for grafting the second most important coronary artery. Due to its ease of harvest and handling, the saphenous vein (SV) continues to be the most commonly used graft for the second most important coronary artery worldwide and, in spite of the mounting evidence^3^, the use of arterial grafts like the radial artery (RA) and right internal mammary artery (RIMA), remains infrequent—accounting for less than 10% of cases in North America^4^.

It appears that, to many, the evidence for more arterial grafts is still not compelling. In order to tackle this problem, the “Radial Artery Patency and Clinical Outcomes” (RAPCO) trial was initiated in Melbourne^5^.

### The RAPCO trial

The RAPCO trial was a prospective randomized trial, with the intention to treat analysis, on patients below 70 years of age (or 60 if they had diabetes mellitus) designed to compare the 10-year patency of the RA with that of the free right internal thoracic artery (RITA) and the SV. The trial started recruiting in 1996 and completed in 2004.

In the RITA versus RA (RAPCO-RITA) arm of the trial, around 400 patients were randomized to receive either RA or free RITA on the second most important coronary target. In the SV versus RA (RAPCO-SV) arm, 225 patients were randomized to receive RA or SV.

The primary endpoint was angiographic graft failure at 10 years, with all-cause mortality being a non-powered, co-primary endpoint outcome. Angiographic graft failure/patency was judged by either coronary angiogram or multi-slice computed tomography (MSCT) of the coronary arteries. The secondary end point was the incidence of major adverse cardiac events, defined as the composite of death, myocardial infarction or repeat coronary revascularization.

In the SV arm, the authors found a trend towards better 10-year patency in the radial artery (85%) versus the saphenous vein (71%) (hazard ratio, 0.40 [95% CI [0.15–1.00]]) and a 10-year survival of 72.6% for the RA group versus 65.2% for the SV (hazard ratio, 0.76 [95% CI [0.47–1.22]]) and no significant difference in the incidence of early complications.

While in the RITA arm; a significant difference in 10-year patency rate in favor of the RA was found (93% versus 83%) (hazard ratio, 0.45 [95% CI [0.23–0.88]]) and survival at 10 years was 90.9% and 83.7% in the RA and RITA groups, respectively (hazard ratio, 0.53 [95% CI [0.30–0.95]])—a trend which was more pronounced in the diabetic sub-group.

## Discussion

The Melbourne group arguably has the highest rate of radial artery usage and, although the trial started 25 years ago, its recently published report comes as a welcome addition to the literature. Between starting the trial and publishing its results, many similar studies were conducted with somewhat inconsistent outcomes.

The SV arm of the RAPCO trial showed a trend towards better patency and survival with RA. This concurs with a meta-analysis of 6 randomised trials, with mid-term follow-up, including the group’s own, concluding that RA had better patency than SV^6^ and with the 2018 European guidelines which support use of RA over SVG in severe stenosis^7^.

The RITA arm of the RAPCO trial showed significantly superior RA patency at 10 years, in contrast with a previous meta-analysis of 8 observational studies showing better patency of both arterial grafts than the saphenous vein^8^. It also contradicts the group’s own large, unadjusted observational study strongly supporting usage of the free RITA^9^. It does, however, partly agree with the large, mutli-centre, arterial revascularization (ART) trial that showed no difference in outcome between RITA and SV^10^.

The investigators were keen to standardize the comparative procedures as much as possible. In order to make the study homogenous, the investigators standardized the surgical setup. All cases were performed on pump with antegrade and retrograde cardioplegia. Only 3 surgeons using the same technique were involved in the study. Additionally, all 3 types of conduits were used as free grafts as an aorto-coronary anastomosis, with exclusion of Y grafts and in-situ RITA. This could have negatively affected the RITA patency as, unlike RA and SV, the thin arterial wall and small lumen of the RITA may not be best accustomed to implantation directly on the ascending aorta.

It is noteworthy that only 65% of the SV group and 80% of the RITA group underwent follow-up imaging, a drawback that has been made up for by a competing risk model where mortality was counted as graft failure. The conclusion was that the RA had significantly higher 10-year patency than free RITA and better than that of SVG and that RA is the free conduit of choice to complement LITA in coronary surgery.

### What have we learned?

The superior results of RA as a free graft are further accentuated by this study. It shows how accumulated experience with use of the radial artery can lead to marked improvements in outcome. This is boosted by the fact that none of the radial arteries in the study have been cannulated for coronary angiogram before, as well as the careful, open harvest technique used, and the choice of target coronary with more than 70% stenosis. In addition, the investigators have systematically applied topical solution for preservation and dilatation of the artery, as well as systemic medication to prevent spasm and improve dilatation after the operation. Together, these technical factors help make the radial artery a superior free graft conduit.

On the other hand, RITA, despite having enhanced histological and biochemical properties, served as an inferior conduit. This can, in part, be explained by the discrepancy in wall thickness and luminal diameter between the ascending aorta and the ITA. Perhaps free RITA as an aorto-coronary conduit is not the best configuration for its long-term patency? It would be interesting to compare the radial artery in its current configuration with an in-situ RITA, or a free graft Y anastomosis from the LITA.

## Conclusion

There are two key clinical take-home from messages from this trial. The radial artery is an excellent conduit if it is used with meticulous technique. It is arguably the best conduit to be used as a free aorto-coronary bypass conduit. Conversely, it is not advisable to use RITA as a free graft from the aorta, but, until further data is available, RITA is far from being written out as the best second conduit for CABG.
